# Supplementary material for: Combinatorial Blood Platelets-Derived circRNA and mRNA Signature for Early-Stage Lung Cancer Detection
Source: Int J Mol Sci. 2023 Mar 2;24(5):4881. doi: 10.3390/ijms24054881 (PMC10003255; doi:10.3390/ijms24054881)
Supplement: Supplementary file 1 [file ijms-24-04881-s001.zip › Supplementary_Files/DAmbrosi et al-Supplementary Tables/Supplementary Table S1.pdf]

Supplementary Table S1.

	Transcript detected	Average counts per transcript	Total count per sample
Control0-48ng	365	850	302910
Control0-24ng	360	362	130618
Control0-12ng	373	148	55390
Control0-6ng	340	90	30893
Control0-3ng	319	73	23551
Control0-1ng	124	61	7605

Supplementary Table S1.

	Transcript detected	Average counts per transcript	Total count per sample
Cancer0-48ng	175	1374	240516
Cancer0-24ng	144	823	118587
Cancer0-12ng	144	340	48993
Cancer0-6ng	121	241	28989
Cancer0-3ng	55	300	16550
Cancer0-1ng	89	84	7527
