# Supplementary material for: Combinatorial Blood Platelets-Derived circRNA and mRNA Signature for Early-Stage Lung Cancer Detection
Source: Int J Mol Sci. 2023 Mar 2;24(5):4881. doi: 10.3390/ijms24054881 (PMC10003255; doi:10.3390/ijms24054881)
Supplement: Supplementary file 1 [file ijms-24-04881-s001.zip › Supplementary_Files/DAmbrosi et al-Supplementary Tables/Supplementary Table S2.pdf]

Supplementary Table S2.

28-mRNA signature	21-circRNA signature	8 mRNA-circRNA signature
IL32	hsa_circRNA_405718	BTK
BTK	hsa_circ_0001535	IRAK2
TNFSF12	hsa_circRNA_104640	PSMB9
HLA-DQA1	hsa_circ_0001380	RUNX1
NFATC2	hsa_circ_0001445	SYK
GBP5	hsa_circRNA_006349	LILRB1 hsa_circ_0000994,
CDKN1A	hsa_circRNA_006296	hsa_circRNA_001937
IGF2R	hsa_circ_0001675	
SERPING1	hsa_circ_0000437	
PSMB9	hsa_circ_0001238	
LAIR1	hsa_circ_0000994	
AHR	hsa_circRNA_101833	
FKBP5	hsa_circ_0005962	
HLA-DMA	hsa_circRNA_100421	
PTPRC_all	hsa_circ_0002360	
LCP2	hsa_circRNA_001937	
TYK2	hsa_circ_0043256	
RUNX1	hsa_circRNA_101367	
JAK3	hsa_circRNA_406483	
CD19	hsa_circRNA_006411	
RAF1	circRNA_HIPK3	
CD59		
SYK		
NCAM1		
MBP		
LILRB1		
IL8		
STAT5B		
