# Supplementary figures and images for: Combinatorial Blood Platelets-Derived circRNA and mRNA Signature for Early-Stage Lung Cancer Detection
Source: Int J Mol Sci. 2023 Mar 2;24(5):4881. doi: 10.3390/ijms24054881 (PMC10003255; doi:10.3390/ijms24054881)

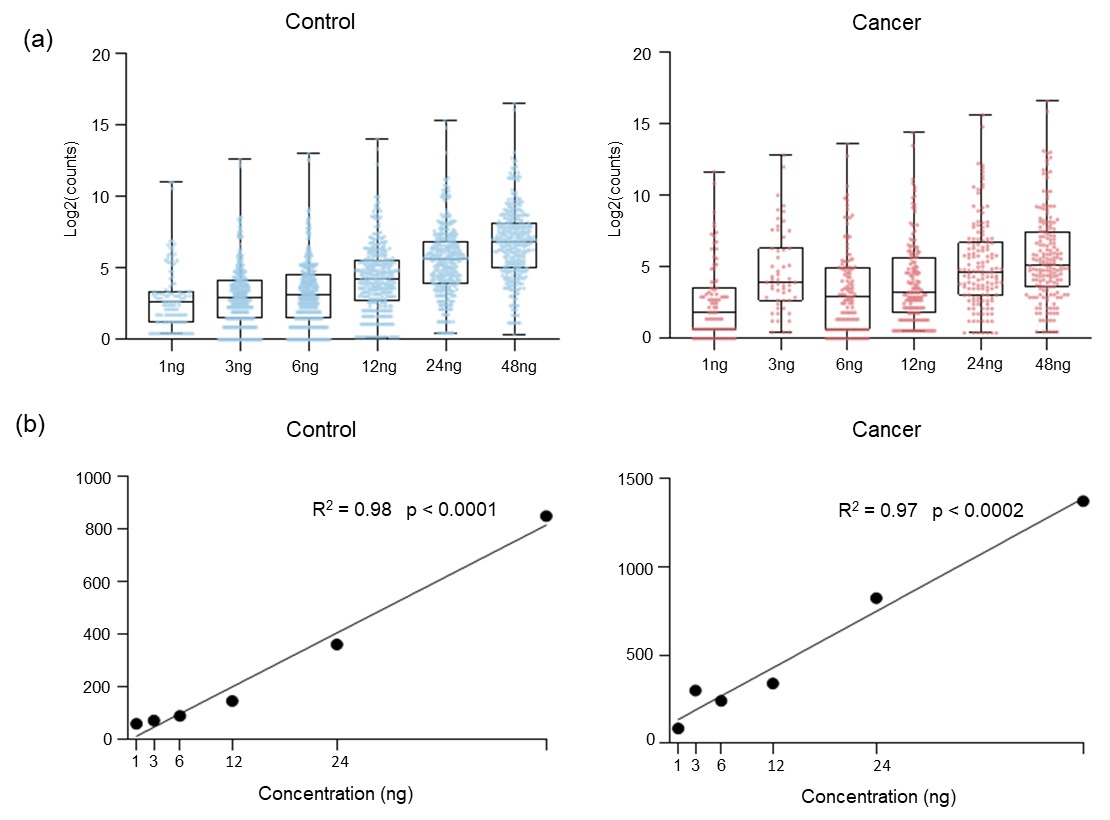

Supplement: Supplementary file 1 [file ijms-24-04881-s001.zip › Supplementary_Files/DAmbrosi et al-Supplementary figures/Supplementary Figure S1.jpg]

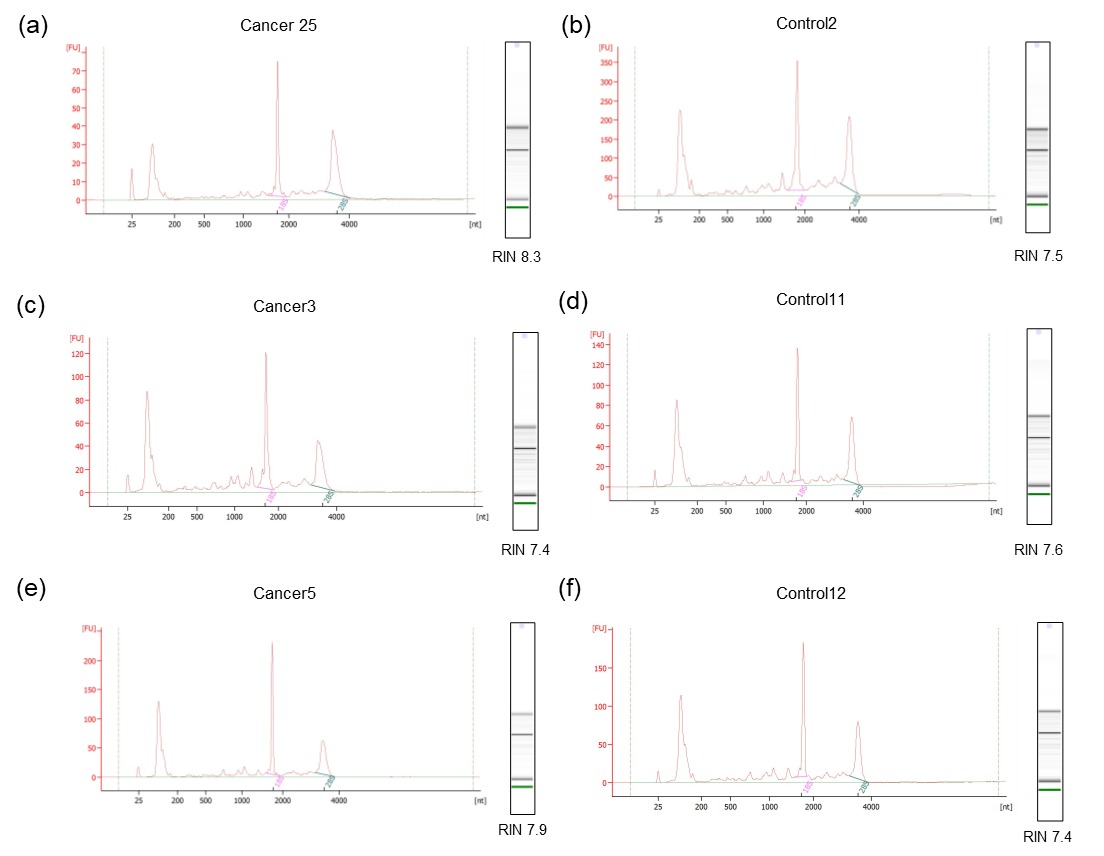

Supplement: Supplementary file 1 [file ijms-24-04881-s001.zip › Supplementary_Files/DAmbrosi et al-Supplementary figures/Supplementary Figure S2.jpg]

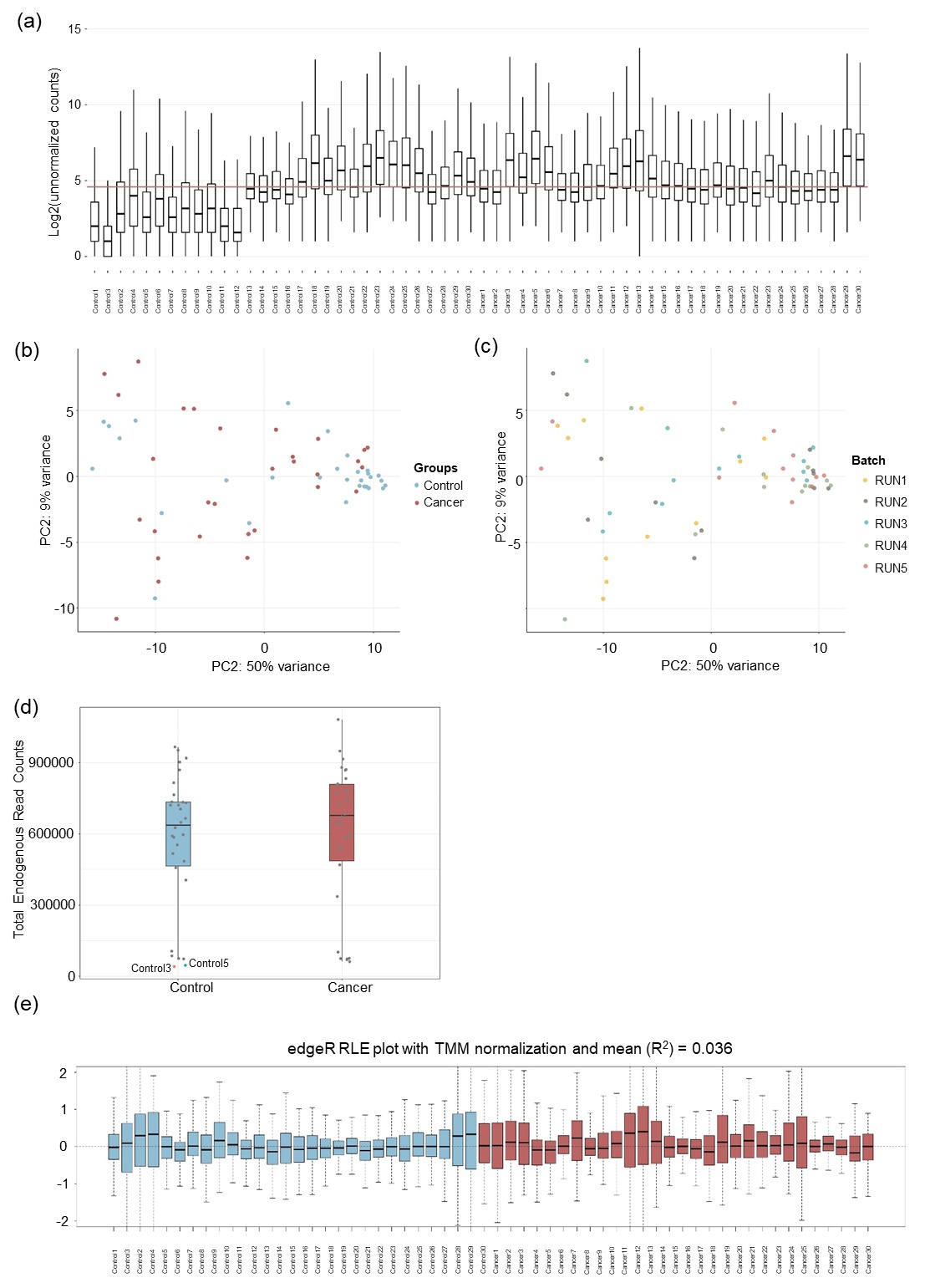

Supplement: Supplementary file 1 [file ijms-24-04881-s001.zip › Supplementary_Files/DAmbrosi et al-Supplementary figures/Supplementary Figure S3.jpg]

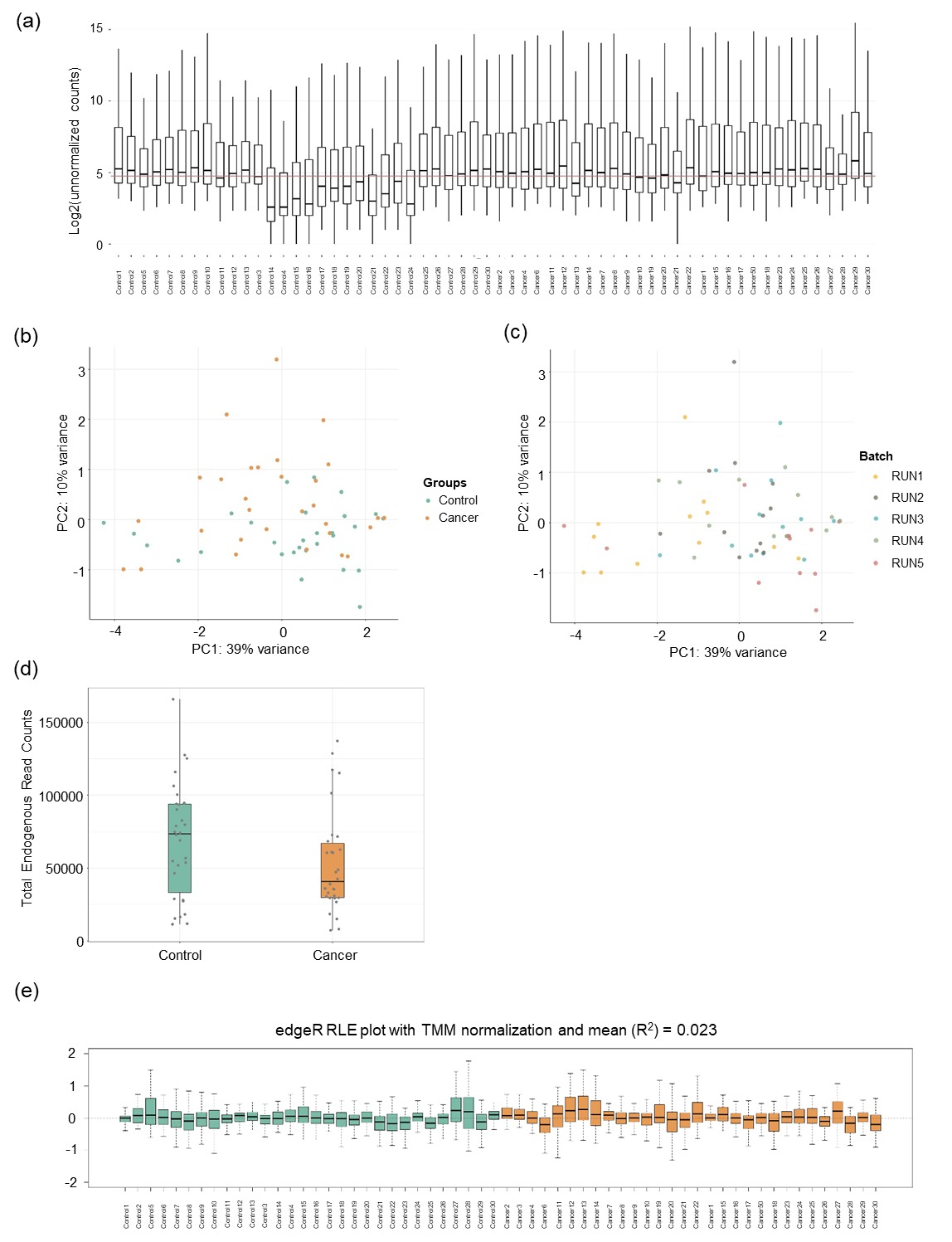

Supplement: Supplementary file 1 [file ijms-24-04881-s001.zip › Supplementary_Files/DAmbrosi et al-Supplementary figures/Supplementary Figure S4.jpg]

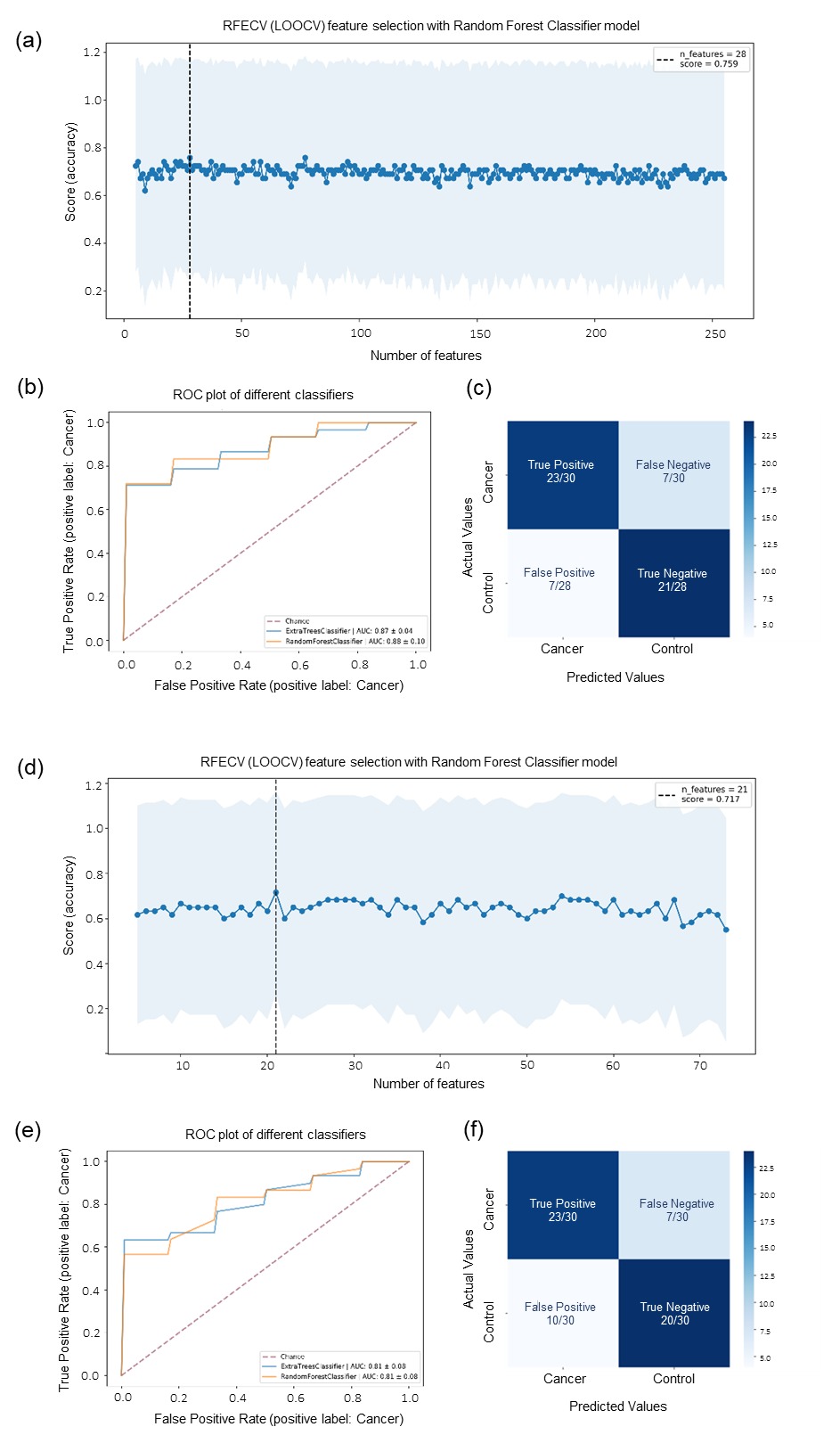

Supplement: Supplementary file 1 [file ijms-24-04881-s001.zip › Supplementary_Files/DAmbrosi et al-Supplementary figures/Supplementary Figure S5.jpg]

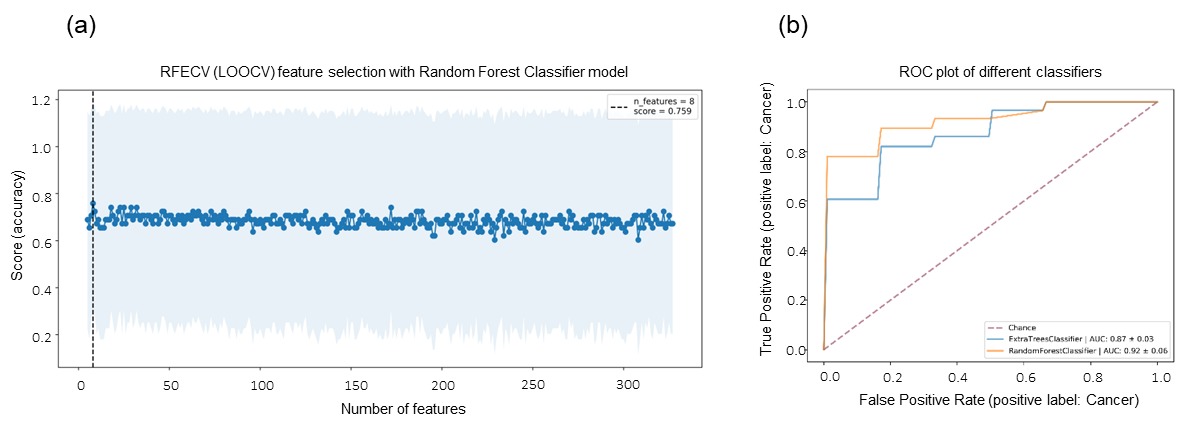

Supplement: Supplementary file 1 [file ijms-24-04881-s001.zip › Supplementary_Files/DAmbrosi et al-Supplementary figures/Supplementary Figure S6.jpg]

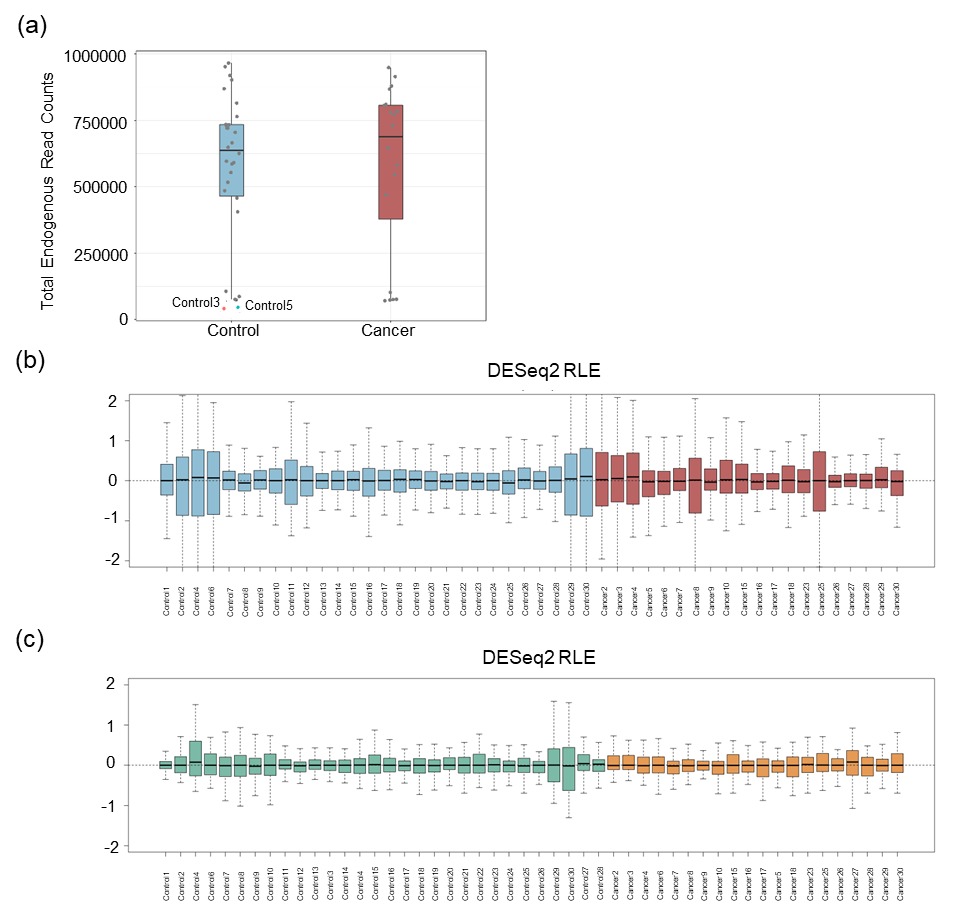

Supplement: Supplementary file 1 [file ijms-24-04881-s001.zip › Supplementary_Files/DAmbrosi et al-Supplementary figures/Supplementary Figure S7.jpg]

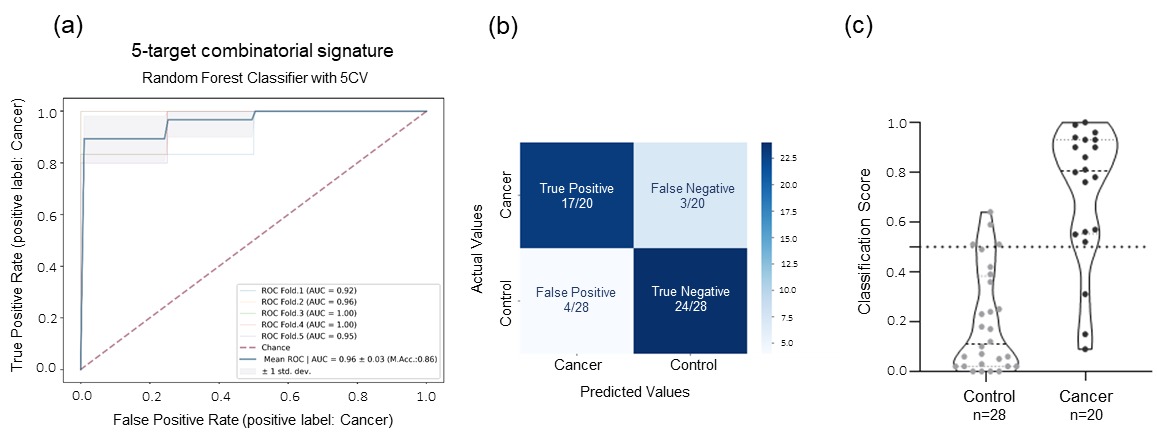

Supplement: Supplementary file 1 [file ijms-24-04881-s001.zip › Supplementary_Files/DAmbrosi et al-Supplementary figures/Supplementary Figure S8.jpg]
